# Evaluation of Central Auditory Processing of Azeri-Persian Bilinguals Using Dichotic Listening Tasks in First and Second Languages

**Published:** 2019

**Authors:** Jamileh FATTAHI, Ali Akbar TAHAEI, Hassan ASHAYERI, Ghassem MOHAMMADKHANI, Shohreh JALAIE

**Affiliations:** 1 Department of Audiology, School of Rehabilitation Sciences, Iran University of Medical Science, Tehran, Iran.; 2 Department of Audiology, School of Rehabilitation, Tehran University of Medical Science, Tehran, Iran.; 3 Department of Basic Sciences, School of Rehabilitation Sciences, Iran University of Medical Science, Tehran, Iran.; 4 School of Rehabilitation, Tehran University of Medical Sciences, Tehran, Iran.

**Keywords:** Bilinguals, Central auditory processing, Dichotic digit test, Reliability, Right ear advantage

## Abstract

**Objectives:**

Sometimes there is no hearing impairment, but it is possible to have an auditory disorder. This is known as a central auditory processing disorder (CAPD). Speech dichotic tasks are useful tools to evaluate CAPD, but there is almost no tool to assess this for Azeri people in their native language. The aim of this study was to evaluate central auditory processing of Azeri participants by Azeri dichotic digit test (ADDT).

**Materials & Methods:**

Participants were 52 normal Iranian Azeri students (mean age 23.27± 4.71; 26 females, 26 males) in the Department of Audiology, School of Rehabilitation Sciences, Iran University of Medical Science, Tehran, Iran in 2016. They were chosen by convenient sampling. ADDT was constructed and administered in free recall conditions along with a Persian dichotic digit test (PDDT). After two to four weeks, reliability was performed.

**Results:**

The mean of the right ear score of PDDT and ADDT was 98.90% and 99.09%, respectively. ADDT was reliable in almost all scores. There was no significant difference in performance between men and women in any score of both tests (*P*>0.05). The results showed a significant difference between both ears’ scores in PDDT (*P*<0.02) as well as in ADDT (*P*=0.00). The right ear advantage was seen in both tests.

**Conclusion:**

All participants performed significantly better on digits presented in the right ear than the left ear in both tests. Central auditory processing of Azeri participants for Azeri is similar to that for Persian.

## Introduction

Central auditory processing (CAP) is the perceptual processing of auditory signals in the central nervous system (CNS) and the neurobiological mechanisms which are substraiting of electrophysiological auditory potentials. CAP encompass sound localization and lateralization; sound discrimination; auditory pattern recognition; temporal characteristics of auditory signals. Central Auditory Processing Disorder (CAPD) is problems with the perceptual processing of auditory signal in the CNS, which reflects by deficiency in one or more of the above mentioned central auditory behaviors (1). Children with CAPD have normal hearing sensitivity and intelligence, but they have some problems with perception of speech in hard listening tasks. The minimum prevalence of CAPD is about 3% to 7% of the population (2), while a prevalence of about 20% has been reported for the students in the United State schools (3). However, children with CAPD typically have no evidence of neurological disease and the diagnosis is made on the basis of performance on behavioral auditory tests (4). Verbal materials used in the tests of CAPD (5). 

Dichotic listening procedure was introduced in 1954, in which the competing pairs of digit series are presented simultaneously into the two ears (6). The following studies, conducted using this procedure, demonstrated its potency in exploring both normal and pathologic function of the brain by auditory stimuli in adults. Dichotic digit test (DDT) includes series of most familiar digits presented to both ears at the same time (7). Normal right-handed individuals obtain greater magnitude of the right ear score compared to the left ear score by using linguistic stimuli in dichotic listening. This phenomenon is called the Right Ear Advantage (REA). The REA for verbal stimulus described in this way that right and left ears are connected to the left and right brain hemispheres, respectively. The contralateral pathways make these connections. Left brain hemisphere is dominant for language processing. The contralateral pathways are more premier than the ipsilateral pathways which organize the link between the ear and the hemisphere on the same side. These explanations of the ear advantages in dichotic listening has been known as the structural hypothesis (8, 9).

DDT is a fast and simple test, developed to evaluate central auditory processing system in dichotic conditions (10, 11) in both adults and children. This test is sensitive to various central auditory system defects such as; brainstem lesions, cortical and sub-cortical disorders (11), and interhemispheric abnormalities. Sensitivity and specificity of DDT are high (10, 11). DDT is not affected by mild to moderate high-frequency loss (12). DDT is described by three scores, including the right ear score, the left ear score, and the right ear advantage (11). Clinical interpretation of dichotic tests depends on REA score. Several studies have used DDT, as a screening tool or part of a test battery, for identification of central auditory processing disorders (7, 8, 10). Large asymmetry between the two ears may be a sign of a processing disorder usually linked to the left ear as a left-ear deficit (13). For this purpose, some training examinations named “Dichotic listening therapy” were performed (14), and were used for rehabilitation of some disorders like learning disorders, dyslexia, and autism (15).

A study was conducted at the development and standardization of Persian double PDDT on 81 normal Persian adults aged 18 to 32 in Iran and found that DDT was a useful tool to assess the auditory processing system in Persian adults (16). A research was performed using Persian double PDDT on 200 normal Persian children in Iran. The results revealed DDT is appropriate to check auditory processing system in children aged 7 to 11 yr (17). Mukari et al. performed a research to construct Malay DDT and administered it to 120 normal Malay children in Malaysia, where their results showed appropriateness of DDT (11, 18). Randomized dichotic digits test has been adapted for using in the Persian language and its test-retest reliability and inter-list equivalency was confirmed (19).

Today, the number of bilingual speakers is more than monolingual speakers in the world's population (20). The first language or mother tongue is called L1 and the second language or foreign language is called L2 (21). With regard to language acquisition, bilinguals may be classified as simultaneous, early and late (21). Bilingualism is a widespread phenomenon in Iran and is not limited to a certain area. About 24% of Iran's population speaks Azeri (22).

DDT is available in different forms, including single, double and triple (23). The Persian versions of single DDT (24), double DDT (16, 17, 25) and randomized DDT are available (18,19) and they are widely used in clinical and research purpose (26-31), but there is no possibility of examination of central auditory system in Azeri people by DDT with their native language. It is necessary to assess central auditory processing of children as soon as possible in childhood. Therefore, it is required to have access to screening tests of Azeri language. Adults were selected as the participants of the present study because the development of dichotic tests is done first in adults (7, 13, 18, 19, 23), and the results are generalized between ages of 12 toward adolescence. The development of dichotic tests in children is fundamental to find its basic norm values in aged 7 to 11 yr (12, 17, 24, 25).

In this study, double Azeri Dichotic Digit Test (ADDT) was constructed and administered along with a double Persian Dichotic Digit Test (PDDT) for Azeri people to study their central auditory processing characteristics. The right ear score, the left ear score and the REA of each test were calculated and analyzed and the results of the PDDT were compared with the results of the ADDT. The objective of the present study was evaluation of central auditory processing of Azeri-Persian bilinguals with dichotic listening tasks in both Azeri and Persian stimuli. 

## Materials & Methods

This cross-sectional comparative study was conducted with feasibility and reliability measuring tools which included two main steps of construction and administration.

## Construction

The Persian stimuli in digit level are currently available and include twenty unique double sets of nine 1-10 monosyllabic digits (16, 17). Since the monosyllabic digits below 10 in Azeri language are limited, we used three digits above 10, consisting of Qırx [40], Yüz [100] and Min [1000]. These digits are not below 10, but they are frequently used. Therefore, eight monosyllable Azeri digits including the Bir (one), Üç (three), dörd (four), Beş (five), On (ten), Qırx (forty), Yüz (hundred), Min (thousand) were utilized to construct ADDT.

Recording of digits was done in a studio by an Azeri-Persian bilingual broadcaster. He had a clear and standard Tabrizi accent and repeated each digit six times with an interval of two seconds with equal tonality and intensity. After completing the recording, the recorded materials were written on CD at 8x by an SBW-06D2X-U CD ROM (ASUS).

Digits were offered through the left and the right channels of the device and they were edited by Cool Edit Pro v2.0 software and the intensity of signals was adjusted on 0VU±1 ^. ^Six seconds of silence between each item was considered for the subject's response. Each item consists of four monosyllabic digits presented to the ears simultaneously. No digit was repeated in an item and each digit had a relatively equal chance to be selected. Between two digits a 500 ms interval was considered. The signals were arranged in such a way that the onset of stimulation was simultaneous to the onset of the signal and a silence interval was considered at the end of each signal to make the duration of each pair of digits was equal Detailed assessment was carried out to ensure that the onset and offset of stimuli were equal. The resulting wave was written with Wave format. At the beginning, a calibration tone with 30 sec duration and frequency of 1000 Hz was recorded on a CD with intensity equal to the average intensity of digits (7, 16, 32).

Twenty-seven different items were made, where each item included four digits. Pair digits were recorded on two separate channels on a CD. In this way, regardless of two training items, the final test was made of 25 items (100 digits) and the total score for each ear was 100%. 

**Ethics consideration**

All participants were selected from Azeri students of Iran University of Medical Sciences (IUMS) and Tehran University of Medical Sciences (TUMS) in Tehran, Iran. 

All participants completed informed consent form. The ethical aspect of this study was approved by the Research Ethics Committee of IUMS in letter 94/1809/105/d. 

**Inclusion criteria: **An Azeri participant was considered as one born in East or West Azerbaijan or Ardabil Provinces of Iran, acquired Azeri language first, but the Persian language (L2) learn relatively early in childhood before the age of five to seven years old and had no interaction with other languages; no history of otologic, neurologic, and audiology disorders, bilateral normal hearing and normal word recognition score; threshold asymmetry less than 15dB (33) (using the Amplaid 311, audiometer) and right handedness assessed through Persian version of Edinburg Handedness Scale. The handedness score ranged from –100 (totally left handed) to +100 (totally right handed).

Hearing assessment and dichotic tests were conducted in Audiology Clinic in the Rehabilitation Science School of IUMS in Tehran, Iran in Oct 2015 until May 2016. Dichotic tests were performed in binaural mode using headphones (Philips SHL 3100 MGY, China). Since all dichotic tests should be implemented at the most comfortable level (MCL) before performing the test, the volume level of the computer was set at 60 dB SPL as the MCL of normal hearing people (33) and calibration was performed in 4152 artificial ears (B&K) using 2235 Sound Level Meter (B&K). Signal presentation conducted via CD player through the laptop using a laptop (Lenovo AMD E1-branded, China). 

At the beginning of each session the device was calibrated and to ensure the leading signal integrity, channel balance, and volume settings, the sound was heard through the headphone, half of the numbers were presented to the right ear and the other half to the left. Both dichotic tests were conducted in free recall format. During free recall, participants were asked to repeat all the digits of both ears in any order (10). Using practice items before the main test ensured the examiner weather participants understood the test. Participants responded to the test orally and each test lasted 4 min. Both tests were carried out on participants randomly to eliminate the sequence effect. Scores of the right ear, the left ear and the difference between two scores (REA) of PDDT and ADDT were obtained. After two to four weeks the re-test was conducted on 33 participants with the same conditions. All tests were done by main researcher.

**Statistical Analysis**

The Kolmogorov-Smirnov test was used for comparing the data distribution against the normal distribution. Spearman correlation coefficient was used to assess the reliability. We Utilized paired t-test to compare the two test scores between the test and the re-test. Independent t-test was used to investigate the effect of gender on the results of each test. We used paired t-test to compare both test scores between both ears to evaluate the ear effect. The data were processed in SPSS 21 (Chicago, IL, USA) at the significance level of 0.05.

## Results

**Figure 1:**
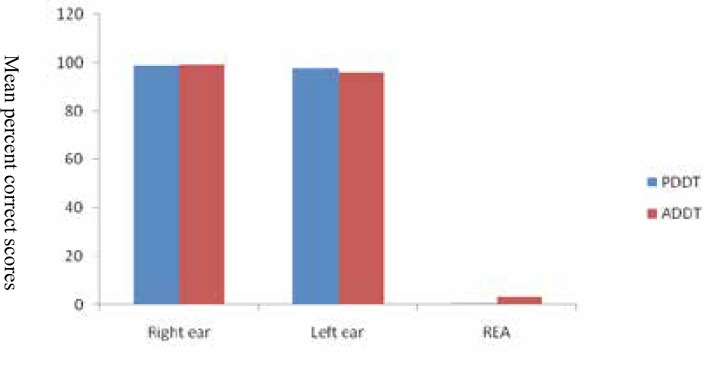
Mean percent correct recognition scores for the right and left ears, and the mean right ear advantage of PDDT and ADDT

Participants were 52 Azeri students (26 female, 26 male). Frequency descriptive of gender of all participants was seen in Figure 1. The range of age was 18 to 35. The means of pure tone average of right and left ears were 4.33 dBHL. The mean lateral preference value based on Edinburgh Handedness Scale was 9.01±1.74. The amounts of mean, Standard Deviation, median, minimum, maximum of scores for both tests in test and re-test are summarized in Table 1. Right ear scores of PDDT and ADDT in first run were 98.90% and 99.09%, respectively. REAs were observed in the two tests, although the values of REAs were not the same. Mean REA score of ADDT (3.41±4.43) was greater than mean REA score of PDDT (0.82±2.5). The retest scores were better than the test scores in both tests.

Test-retest reliability was surveyed by comparing the mean scores of the first and the second run. All scores of both tests were reliable (*P*>0.05) with the exception of the left ear score of ADDT (*P*=0.02). To investigate the reliability of both tests, Spearman correlation coefficient was also used. Total scores in bilinguals were repeatable (*P*<0.05), but the right ear score of ADDT and the REA score of PDDT were not reproducible (Table 2). The analysis of the data for determining the effect of gender on results can be seen in Table 3. In both tests, there is no significant difference in performance between men and women in any score of PDDT and ADDT (Table 3). The impact of ear effect on PDDT results revealed a significant difference between the scores of right and left ears for Persian stimuli (*P*=0.019). Significant difference between the two ears confirmed an REA in PDDT. The comparison of the scores of right and left ears for ADDT in bilinguals also showed a highly significant difference (*P*=0.001). The obtained value approved a significant REA in Azeri stimuli.

## Discussion

This study was aimed to construct and assess the ADDT and PDDT. REAs exist in both Persian and Azeri stimuli in bilingual speakers, although the values of REA were not the same (Figure 1). The mean of the right ear, the left ear and REA scores for PDDT in the present study (98.9, 98.03 and 0.82) are consistent with another result (17). Their results of PDDT for the above scores were respectively 100± 0.00, 98.412.02± and 1.59± 2.02 (17). Furthermore, the mean of right ear score of PDDT (98.90%) is comparable to ADDT (99.09%) and previous studies (7, 12, 17, 34). 

**Reliability**

The reliability was assessed using correlation coefficients and paired t-test. Total scores based on Spearman correlation coefficient were repeatable but the right ear score of ADDT and REA score of PDDT were not reproducible. In this way, four out of six calculated scores showed repeatable results. Results are reliable (Table 2). 

**Table 1:** Descriptive measures of all scores for PDDT and ADDT in test and re-test and (n=52)

		PDDT			ADDT		
		Right ear	Left ear	REA	Right ear	Left ear	REA
Test	Mean	98.90	98.03	0.82	99.09	95.67	3.41
	SD	1.60	2.94	2.5	2.04	4.06	4.43
	Median	100	100	0.00	100	97.5	2.5
	Minimum	95	87.5	-2.5	92.5	85	-5
	Maximum	100	100	10	100	100	15
Re-test	Mean	99.47	99.01	0.45	99.54	96.97	2.42
	SD	1.62	1.76	2.02	0.98	3.47	3.22
	Median	100	100	0.00	100	97.50	2.50
	Minimum	92.5	92.5	-7.5	97.5	85	-2.5
	Maximum	100	100	5	100	100	12.5

PDDT =Persian dichotic digit test

ADDT = Azeri dichotic digit test

REA= Right ear advantage

SD=standard deviation

PDDT =Persian dichotic digit test

ADDT = Azeri dichotic digit test

REA= Right ear advantage

**Table 2:** *P-*values of test-re test reliability and Spearman’s correlation test between test-retest for ADDT and PDDT (n=52)

	Scores		Reliability
	test-re test		Spearman’s correlation
		*P*-value	*P*-value*
ADDT	Right ear	0.09	0.114
	Left ear	0.02	0.000
	Ear advantage	0.23	0.003
PDDT	Right ear	0.11	0.001
	Left ear	0.08	0.006
	Ear advantage	0.50	0.732

PDDT =Persian dichotic digit test

ADDT = Azeri dichotic digit test

REA= Right ear advantage

**Table 3:** Mean (standard deviation) the scores of right ear, left ear and right ear advantage of PDDT and ADDT in total population and comparison of the scores in men and women

Test	Scores	Gender	mean	standard deviation	*P* value
PDDT	Right ear	female	98.85	1.62	0.83
		male	98.94	1.61	
	Left ear	female	97.98	3.16	0.91
		male	98.08	2.77	
	REA	female	0.77	2.53	0.89
		male	0.87	2.54	
ADDT	Right ear	female	99.04	2.01	0.87
		male	99.13	2.11	
	Left ear	female	95.29	4.02	0.50
		male	96.06	4.13	
	REA	female	3.75	4.02	0.59
		male	3.08	4.86	

Legend:

PDDT =Persian dichotic digit test

ADDT = Azeri dichotic digit test

REA= Right ear advantage

Test-retest reliability indicated that five out of six calculated scores are repeatable. Thus, almost all results are reliable. Reliability of test-retest can somehow show the learning effect on the results. There was no learning effect on any score of any tests except the left ear score of ADDT (Table 2). Left ear performance for Azeri stimuli was different from that of the right ear. In the present study, we consider the interval of two to four weeks between the first and second run like previous study (17). This interval between two run tests was probably not appropriate for bilinguals.

**Gender effect**

In the present study, the gender did not affect the results of PDDT and ADDT. This finding is in agreement with other studies (35-38) in which both sexes showed analogous performance. However, there is a disharmony with earlier studies that reported a significant sex difference in the magnitude of laterality effects (39). 

**ADDT vs. PDDT:**

The results of the present study clearly indicated REAs for PDDT and ADDT, presented stimuli were processed in the left hemisphere. This finding is in line with the several previous studies (8, 10-12, 17, 23-25), confirming the dominance of the left hemisphere for speech and language perception in normal right-handed listeners in dichotic listening. We found clearly indicated REAs (the left hemisphere dominance) for Azeri stimuli (L1) presented in Azeri participants by dichotic listening tasks. This finding was also reported in Cantonese–English (40), English-French (41), and Portuguese-English (42). 

Greater REA of Azeri (L1 compared to REA of Persian (3.41 vs. 0.82), is the inequality of the left ear scores (95.67 vs. 98.03) and similarity of the right ear scores (99.09 vs. 98.90). Increasing the left ear score and decreasing the left ear score in Persian are indicative of the effect of bilingualism on results. The findings of the present study showed different REAs for Azeri and Persian stimuli, this finding was also reported in Chinese-English (43) and Italian -English (44) bilinguals in dichotic listening task conditions. Our results on the study of lower REA value in L2 (Persian) compared to L1 (Azeri) are in line with other findings. Lower degree of hemispheric lateralization found for L2 than for L1 in an ERP study on simultaneous interpreters (45). The results of the present study regarding REA/LH dominance both in L1 and L2 are in contrast with the study using the orthographic task (46). They reported an absolute symmetrical pattern for the simultaneous interpreters both in L1 (Italian) and L2 (English). There is a difference between two studies. In the present study participants were Azeri-Persian bilinguals evaluated by dichotic listening tasks, but participants were Italian-English simultaneous interpreters assessed by the orthographic task. The differences are likely related to the age, proficiency level of L2 and methods of acquisition. Although symmetrical pattern results of L2 in Proverbio ’study is in line lower REA results of L2 in present study (46). 

Since dichotic test results do not change after the age of 12 (9-12), the results of the present study can be generalized in such a group age of children and adults. In this study, central auditory screening test was made available and caused the possibility of the assessment of Azeri people in their native language and, led to the facility of the assessment and rehabilitation of disorders like learning disorders, dyslexia, autism, and split brain. Future studies are conducted on Azeri speakers to develop lists of ADDT and perform test on larger samples and wider age range, particularly children before the age of 12. Furthermore, these tests are conducted with the different intervals between test-retest to obtain better reliability data. Moreover, the validity of ADDTs in identifying auditory processing disorders could be studied by testing participants with suspected auditory processing deficits such as children with learning disability, dyslexia, autism, and adults with known hemispheric pathology. This study is replicated in children for tracking maturation trends in the ear scores for the first language and the second language.

**In conclusion, **Azeri version of double DDT is a reliable tool in almost all scores. Gender has no effect on any score of ADDT. Although the values of REA for PDDT and ADDT were not the same, REAs were observed for both ADDT and PDDT. Using ADDT to assess central auditory processing of Azeri people in dichotic listening seems to be useful.
